# The Association Between Changes in Plasma Short-Chain Fatty Acid Concentrations and Hypertension in Children With Chronic Kidney Disease

**DOI:** 10.3389/fped.2020.613641

**Published:** 2021-02-04

**Authors:** Pei-Chen Lu, Chien-Ning Hsu, I-Chun Lin, Mao-Hung Lo, Ming-Yu Yang, You-Lin Tain

**Affiliations:** ^1^Department of Pediatrics, Kaohsiung Chang Gung Memorial Hospital and College of Medicine, Chang Gung University, Kaohsiung, Taiwan; ^2^Graduate Institute of Clinical Medical Sciences, College of Medicine, Chang Gung University, Taoyuan, Taiwan; ^3^Department of Pharmacy, Kaohsiung Chang Gung Memorial Hospital and College of Medicine, Chang Gung University, Kaohsiung, Taiwan

**Keywords:** children, chronic kidney disease, hypertension, short-chain fatty acid, acetate, propionate, butyrate

## Abstract

**Background:** Some children with chronic kidney disease (CKD) develop hypertension faster than others. This may be attributable to endothelial dysfunction, among other reasons. Short-chain fatty acids (SCFAs), that is, acetate, butyrate, and propionate, are known for reducing cardiovascular risks *via* preserving endothelial function. This study aimed to investigate the association between changes in plasma SCFA concentrations and in cardiovascular and endothelial parameters in children with CKD.

**Methods:** In total, 105 children and adolescents who met the CKD criteria were enrolled in this study, and 65 patients aged >6 years were divided into two groups based on the ambulatory BP measurements. The parameters of plasma SCFAs, endothelial function and morphology, and echocardiography were examined at the index visit and followed up after 1 year.

**Results:** We observed that 27.69% of 65 patients developed hypertension during the study period. Plasma acetate increased by 22.75 μM in the stable group (*P* < 0.001), whereas there was no change in the worsened BP group. The index higher plasma butyrate was positively correlated with worsened BP (adjusted odd ratio, 1.381; *P* = 0.013). At the follow-up, plasma butyrate decreased by 2.12 and 4.41 μM in the stable and worsened BP groups, respectively (*P* < 0.001). In 105 subjects, higher index plasma propionate was positively correlated with decreasing ejection fraction (adjusted odd ratio, 1.281; *P* = 0.046).

**Conclusions:** Plasma acetate seemed to play a role in preventing hypertension in children with CKD. However, the index plasma propionate and butyrate concentrations seemed to imply the development of cardiovascular problems in our 1-year study.

## Introduction

Children with chronic kidney disease (CKD) are at high risk for the accelerating development of cardiovascular disease (CVD). They may not have any perceptible symptoms during childhood, but certain parameters regarding endothelial dysfunction, that is, increased carotid artery intima-media thickness (IMT) and increased pulse wave velocity (PWV), are frequently observed in these children (1, 2). However, hypertension develops faster in some children with CKD compared with others, which is primarily attributed to the progression of CKD; however, deterioration of kidney function is not observed in all children with CKD and hypertension.

Hypertension is a complex phenomenon associated with endothelial dysfunction. Recent studies have reported that short-chain fatty acids (SCFAs) have inflammatory effects and preserve endothelial function (3). SCFAs are saturated aliphatic organic acids with fewer than six carbon atoms and are generated from microbial fermentation of partially and non-digestible polysaccharides in the colon. Acetate, propionate, and butyrate are the most abundant SCFAs (≥95%) (4), which are detectable in the peripheral blood. SCFAs are generally considered beneficial to blood pressure (BP) regulation. Decreased SCFA concentrations were reported in a mouse model of metabolic syndrome (5) and in kidney disease (6). Increased dietary fiber intake, which is expected to increase plasma SCFA concentrations, is also associated with decreased blood pressure (7). Therefore, we measured plasma SCFA concentrations in children and adolescents categorized into two groups based on BP levels. Higher SFCA concentrations after1 year in normotensive subjects compared with hypertensive subjects would suggest a potential approach for the prevention of cardiovascular disease and implicate that proper SCFA consumption *via* high-fiber diet or SCFA supplementation can slow the progression of hypertension in children and adolescent with CKD.

However, higher SCFA concentrations were also observed in some unexpected situations. Our previous study found that children with CKD and an abnormal ambulatory BP monitoring (ABPM) profile had higher plasma concentrations of propionate and butyrate (8). Certain diseases linked to poor BP control, such as diabetes mellitus and obesity, were found to have drastic increase in circulating propionate concentrations (9, 10). These findings are in contrast with current perceptions. We aimed to investigate the association between plasma SCFA concentrations and the changes in cardiovascular parameters in children with CKD. Thus, we conducted an observational clinical trial including children with CKD with and without hypertension. We aimed to test our hypothesis that children with CKD who develop hypertension faster than others would have lower plasma SCFAs and that low plasma SCFA concentrations were associated with other cardiovascular changes.

## Methods

### Patients and Study Design

The retrospective cohort study was in accordance with the principles of the 1964 Declaration of Helsinki and its later amendments. Written informed consent was obtained from all patients before participating in this study. Children and adolescents aged 3 to 18 years with CKD from the pediatric nephrology outpatient clinics in Kaohsiung Chang Gung Memorial Hospital, Taiwan, were recruited between December 2016 and October 2018. CKD was defined and staged according to the National Kidney Foundation's Kidney Disease Outcomes Quality Initiative (K/DOQI) (11). Using the bedside CKiD equation, kidney function was expressed as estimated glomerular filtration rate (eGFR) based on height and serum creatinine concentration (12).

A patient was excluded from this study if she/he (a) had eGFR <15 ml min^−1^ 1.73 m^−2^, (b) was undergoing dialysis, (c) was a kidney transplant patient, (d) was currently pregnant, (e) had a history of congenital heart disease, (f) lost to follow-up, or (g) was unable to cooperate during the examinations.

The analysis was limited to children and adolescents with baseline eGFR >15 ml min^−1^ 1.73 m^−2^. The measurements of renal and cardiovascular parameters are described in the following sections.

### Data and Specimens

The following examinations were performed on a subject at a clinic visit: (a) history taking, office BP measurements, and physical examination; (b) blood and urine investigations; (c) echocardiography; (d) ABPM; and (e) carotid sonography. The etiologies of kidney diseases were divided to two categories: (a) congenital anomalies of kidney and urinary tract (CAKUT) and (b) non-congenital anomalies of kidney and urinary tract (non-CAKUT). CAKUT includes renal hypoplasia, renal dysplasia, unilateral renal agenesis, reflux nephropathy, polycystic kidney disease, horseshoe kidney, duplex kidney, posterior urethral valve, and ureter abnormalities.

### Anthropometric Measurements

Body mass index (BMI) z-score was based on data from the Health Promotion Administration, Ministry of Health and Welfare in Taiwan (13).

### Office BP and ABPM Measurements

According to the 2017 American Academy of Pediatrics (AAP) guidelines (14), hypertension was diagnosed as systolic blood pressure (SBP) or diastolic BP (DBP) ≥95th percentile or 130/80 mmHg for subjects aged 1–13 years or SBP or DBP ≥130/80 mmHg for subjects older than 13 years old. After sitting for 5 min, office BP was recorded from the subjects using a verified electronic sphygmomanometer.

Data from 24-h ABPM were collected from subjects aged 6–18 years within 1 week after clinic visits, using the Oscar 2 ABP Monitor (SunTech Medical Inc., Morrisville, NC, USA). BP and heart rate (HR) were recorded by ABPM every 20 min for 24 h, while these subjects were normally active. BP load was defined as the percentage of hypertensive BP readings. The second timing of ABPM, as well as other exams, was approximately 1 year later.

The criteria of hypertension diagnosed by 24-h ABPM included (a) mean daytime SBP/DBP exceeding the 95th percentile or 130/80 mmHg and (b) mean night-time SBP/DBP dropping <10% below 95th percentile or 130/80 mmHg.

### The Definition of Worsened BP and Stable BP

Based on two-time ABPM data in the index and the following visits, the category of worsened BP group included both patients with persistent and worse BP, and stable BP includes both no hypertension and improved BP on follow-up.

### Cardiac and Vascular Assessments

Echocardiography and carotid ultrasound were scheduled at the same visit of BP measurements. Echocardiography was performed by pediatric cardiologists (I-Chun Lin, Mao-Hung Lo). Based on the 2017 AAP guideline, left ventricular mass index (LVMI) was calculated as left ventricular (LV) mass (g) divided by height (m) to the 2.7th power (14). The report recommends LV hypertrophy (LVH) to be defined as LVMI >51 g m^−2.7^ (14). Low ejection fraction (ejection fraction) was defined <53% (14).

Carotid sonography was performed by I-Chun Lin, Mao-Hung Lo, and Pei-Chen Lu as previously reported (8). A 5–12-MHz linear array transducer was coupled with ProSoundα7 ultrasound and computer-aided analysis software (eTRACKING system; Aloka Co., Ltd., Tokyo, Japan) to obtain an image of the left common carotid artery. Measurements of cardiovascular abnormalities and arterial stiffness include LV mass, LVMI, EF, carotid IMT, and PWV.

### Gas Chromatography–Flame-Ionization Detector

As previously reported (8), circulating acetate, butyrate, and propionate concentrations were measured using a gas chromatography–mass spectrometer (GCMS-QP2010; Shimadzu, Kyoto, Japan) with flame-ionization detector (FID). Separation was performed on an SGE BP GC column (21 m × 0.5 μm, 30 m × 0.53 mm; Shimadzu GLC Ltd., Tokyo, Japan). The working solutions of acetate, propionate, and butyrate used as internal and external standards at the concentration of 10 mM were kept at 20°C in the refrigerator. Dry air, nitrogen, and hydrogen were fed into FID at 300, 20, and 30 ml min^−1^, respectively. An aliquot of 2 μl sample was injected into the column. The temperatures of inlet and FID were set to 200°C and 240°C, respectively. The total running time was 17.5 min. Analytical standard grades as internal standards for acetate and propionate were acquired from Sigma-Aldrich (St. Louis, Missouri, USA), and internal standards for butyrate from Chem Service (West Chester, PA, USA).

### Statistical Analysis

Continuous variables were expressed as median (25th, 75th percentile), whereas categorical variables were reported as number (%). Non-parametric statistics or Chi-square test was used to compare the index variables between subjects with or without worsened BP. Paired *t* test was used to examine differences in variables between subjects between index and following visits. Multivariate logistic analysis was performed to investigate the associations between plasma concentrations of SCFAs and worsening renal and cardiovascular parameters (worsened BP, increased LV mass, increased LVMI, reduced EF, increased IMT, increased PWV, decreased eGFR). SPSS version 22.0 (SPSS, Inc., Chicago, IL, USA) was used for all analyses.

## Results

**Figure 1:**
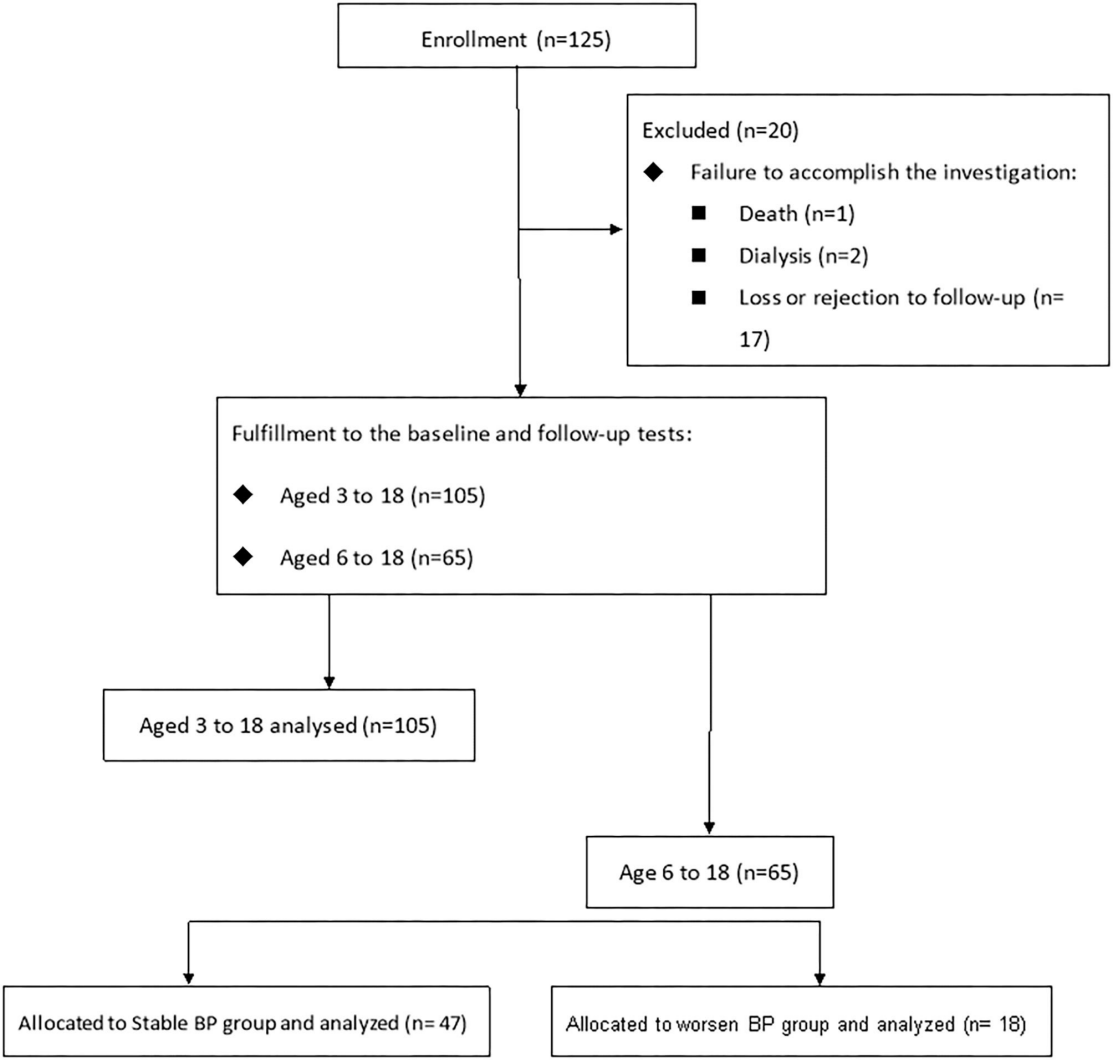
The retrospective cohort study included 105 subjects (aged 3–18) having echocardiographic data, anthropometric measurements, hemogram and blood chemistry profiles, and plasma short-chain fatty acids for analysis. Of them, 65 subjects completing carotid echographic data and ambulatory blood pressure measurements were categorized into two groups based on blood pressure development within 1 year.

The design of study is presented in Figure 1. Data were available for 125 children and adolescents from Kaohsiung Chang Gung Memorial Hospital. Twenty children and adolescents were excluded from analysis due to death secondary to meningitis with underlying spinal bifida and hydrocephalus (1/125, 0.8%), need for dialysis (2/125, 1.6%), and loss or rejection of follow-up (17/125, 13.6%). A total of 105 children and adolescents completed the index and the following medical examinations. Of 105 subjects, 65 had extra data of 24-h ABPM and neck sonography.

### Baseline Clinical Characteristics of the Study Patients

Data are presented in Table 1. Median age was 9.6 years (interquartile range, 5.5, 14.4 years), 57.1% were male, 66.7% had CAKUT as etiology, and all are Taiwanese. The median eGFR was 107.1 ml min^−1^ 1.73 m^−2^ (interquartile range, 88.3, 124.4 ml min^−1^ 1.73 m^−2^); 73.3% had CKD stage G1, 18.1% were G2, 5.7% were G3, and 2.9% were G4.

**Table 1:** Baseline patient characteristics of enrolled 105 subjects.

**Index data**	
*N* = 105	
Age (years)	9.60 (5.50, 14.40)
Male sex	60 (57.1%)
CAKUT	70 (66.7%)
BMI (z-score)	−0.09 (−0.50, 0.70)
Obesity	13 (12.4%)
eGFR (ml min^−1^ 1.73 m^−2^)	107.10 (88.3, 124.4)
UPCR (mg g^−1^)	68.00 (41.50, 256.00)
**CKD stage**	
G1	77 (73.3%)
G2	19 (18.1%)
G3	6 (5.7%)
G4	3 (2.9%)
eGFR >90 ml min^−1^ 1.73 m^−2^ and UPCR <150 mg g^−1^	53 (50.5%)
Office SBP or DBP >95th percentile	36 (34.4%)
Office SBP (mmHg)	109 (100, 119)
Office DBP (mmHg)	67 (62, 77)
LDL-C (mg dl^−1^)	67 (16.5, 82.5)
TG (mg dl^−1^)	163.50 (143.25, 184.75)
Uric acid (mg dl^−1^)	5.0 (4.0, 6.0)

Data are expressed as median (25th, 75th) or n (%).

*BMI, body mass index; CAKUT, congenital anomalies of kidney and urinary tract; CKD, chronic kidney disease; DBP, diastolic blood pressure; eGFR, estimated glomerular filtration rate; LDL-C, low-density lipoprotein cholesterol; SBP, systolic blood pressure; TG, triglyceride; and UPCR, urine protein-to-creatinine ratio*.

Baseline characteristics by presence or absence of worsened BP are presented in Table 2. Eighteen (27.69%) of 65 subjects had worsened BP, whereas BP of 47 (72.31%) subjects remained constant. Of the worsened BP group, more than half were hypertensive at index year, whereas only 12.80% of the stable BP group had hypertension (*P* < 0.001). These worsened BP group were older (15.65 vs. 12.00 years, *P* = 0.015) and more male predominant (83.39 vs. 46.80%, *P* = 0.008) and had higher BMI z-score (0.54 vs. −0.25, *P* = 0.036). Obesity was more prevalent in this group, accounting for closely 40%, whereas only 10.63% were obese in the stable BP group. Only 22.22% of the worsened BP group were CKD stage G1, which is 26.72% lower than the stable BP group (*P* = 0.050). Nearly 40% of the worsened BP group took immunosuppressants for treating their renal diseases and <7% of the stable BP (38.90 vs. 6.4%, *P* = 0.001).

**Table 2:** Baseline clinical characteristics by presence of worsened BP.

**Index data**	**Stable BP group**	**Worsened BP group**	
***N*** **=** **65**	***N*** **=** **47**	***N*** **=** **18**	***P***-value^*^
Age (years)	12.00 (9.60, 14.6)	15.65 (11.38, 16.63)	0.015
Male sex	22 (46.80%)	15 (83.30%)	0.008
eGFR (ml min^−1^ 1.73 m^−2^)	110.40 (85.30, 122.70)	87.85 (75.78, 117.95)	NS
UPCR (mg g^−1^)	58.10 (35.30, 183.60)	117.25 (41.33, 1464.53)	NS
CKD stage G1	23 (48.94%)	4 (22.22%)	0.050
CAKUT	29 (61.70%)	9 (50.00%)	NS
BMI (z-score)	−0.25 (−0.52, 0.52)	0.54 (−0.32,1.53)	0.036
Obesity	5 (10.63%)	7 (38.89%)	0.009
Office SBP or DBP >95th percentile	18 (38.30%)	12 (66.67%)	0.040
24-h ABPM SBP or DBP >95th percentile	6 (12.80%)	10 (55.56%)	<0.001
**24-h ABPM SBP and DBP** **≤95th percentile**			
Receiving anti-HTN drug	5 (10.64%)	4 (22.22%)	NS
Not receiving anti-HTN drug	36 (76.60%)	4 (22.22%)	0.001
Immunosuppressants	3 (6.40%)	7 (38.90%)	0.001
LDL-C >98.5 mg dl^−1^	11 (23.40%)	5 (27.78%)	NS
TG >101 mg dl^−1^	8 (17.00%)	7 (38.90%)	NS
Uric acid >6.0 mg dl^−1^	12 (25.53%)	13 (72.22%)	0.001

Data are expressed as median [25th, 75th] or n (%).

* P < 0.05 by the Chi-square test or Mann–Whitney U test.

*BMI, body mass index; CAKUT, congenital anomalies of kidney and urinary tract; CKD, chronic kidney disease; DBP, diastolic blood pressure; eGFR, estimated glomerular filtration rate; HTN, hypertension; LDL-C, low-density lipoprotein cholesterol; SBP, systolic blood pressure; TG, triglyceride; UPCR, urine protein-to-creatinine ratio*.

### Baseline Cardiovascular Characteristics of the Study Patients

Comparison of the index data of the ABPM profile, echocardiography, carotid echography, and plasma SCFAs by the presence of future hypertension was presented Table 3.

**Table 3:** Baseline cardiovascular characteristic by presence of worsened BP.

**Index data**	**Stable BP group**	**Worsened BP group**	
***N*** **=** **65**	***N*** **=** **47**	***N*** **=** **18**	***P***-value^*^
**ABPM profile**			
Mean SBP overall (mmHg)	109.00 (101.00, 115.00)	126.00 (117.25, 137.50)	<0.001
Mean SBP awake (mmHg)	113.00 (105.00, 119.00)	129.50 (120.25, 137.00)	<0.001
Mean SBP asleep (mmHg)	100.00 (91.00, 108.00)	118.50 (109.75, 129.50)	<0.001
Mean DBP overall (mmHg)	63.00 (58.00, 65.00)	67.00 (64.00, 76.75)	0.001
Mean DBP awake (mmHg)	66.00 (62.00, 69.00)	69.50 (67.00, 77.75)	0.005
Mean DBP asleep (mmHg)	55.00 (51.00, 59.00)	62.50 (55.75, 78.75)	0.001
SBP load overall (%)	2.00 (0.00, 8.00)	35.50 (20.25, 49.50)	<0.001
SBP load awake (%)	2.00 (0.00, 8.00)	23.50 (12.50, 38.25)	<0.001
SBP load asleep (%)	0.00 (0.00, 13.00)	59.00 (28.75, 92.50)	<0.001
DBP load overall (%)	2.00 (0.00, 5.00)	8.00 (2.75, 35.25)	<0.001
DBP load awake (%)	0.00 (0.00, 3.00)	8.00 (2.00, 15.00)	<0.001
DBP load asleep (%)	0.00 (0.00, 6.00)	10.50 (0.00,54.25)	0.007
**Carotid artery**			
IMT (mm)	0.35 (0.30, 0.38)	0.38 (0.34, 0.40)	0.013
PWV (m s^−1^)	3.60 (3.30, 4.10)	4.40 (3.70, 4.63)	0.006
**Cardiac echography**			
LV mass (g)	85.80 (64.85, 116.25)	114.50 (83.40, 177.00)	0.021
LVMI (g m^−2.7^)	29.92 (25.96, 36.30)	33.52 (30.32, 46.19)	0.022
EF (%)	64.80 (62.20, 72.000	66.15 (59.15, 69.35)	
LVH	5 (10.64%)	8 (44.44%)	<0.001
Low EF	0 (0.00%)	0 (0.00%)	NS
**Short-chain fatty acid**			
AA (μM)	29.15 (17.40, 48.20)	30.90 (17.03, 52.15)	NS
PA (μM)	2.20 (1.38, 3.00)	1.60 (1.08, 3.60)	NS
BA (μM)	2.20 (1.20, 4.23)	5.65 (2.08, 8.90)	0.005

Data are expressed as median [25th, 75th] or n (%).

* P < 0.05 by the Chi-square test or Mann–Whitney U- test.

*AA, acetate; ABPM, ambulatory blood pressure monitoring; BA, butyrate; DBP, diastolic blood pressure; EF, ejection fraction; IMT, intima-median thickness; LV, left ventricle; LVH, left ventricular hypertrophy; LVMI, left ventricular mass index; PA, propionate; PWV, pulse wave velocity; and SBP, systolic blood pressure*.

Overall, median BPs were higher in the worsened BP group than the stable BP group (mean SBP overall 126 vs. 109 mmHg; mean SBP awake 129.5 vs. 113 mmHg; mean SBP asleep 118.5 vs. 110 mmHg; mean DBP overall 67 vs. 63 mmHg; mean DBP awake 69.5 vs. 66 mmHg; mean DBP asleep 62.5 vs. 55 mmHg; all *P* < 0.05). Furthermore, in the BP loads, worsened BP group revealed significantly higher values than the stable BP group (SBP load overall 35.5 vs. 2%; SBP load awake 23.5 vs. 2%; SBP load asleep 59 vs. 0%; DBP load overall 8 vs. 2%; DBP load awake 8 vs. 0%; DBP load asleep 10.5 vs. 0%; all *P* < 0.05).

Indicators for cardiovascular deterioration, such as heavier LV mass, higher LVMI, thicker IMT, and higher PWV, were noted in the worsened BP group compared with stable BP group (LV mass 114.50 vs. 85.80 g, *P* = 0.021; LVMI 33.52 vs. 29.92 g m^−2.7^, *P* = 0.022; IMT 0.38 vs. 0.35 mm, *P* = 0.013; PWV 4.40 vs. 3.60 m s^−1^, *P* = 0.006). Eight subjects (44.44%) in the worsened BP group had LVH, whereas five subjects (10.64%) in the stable BP group had LVH (*P* < 0.001).

No statistical difference was found in the plasma acetate and propionate concentrations between the stable and worsened BP groups (*P* > 0.005). Interestingly, plasma butyrate was 5.65 μM in the worsened BP group, which was 3.45 μM higher than that in the stable BP group (*P* = 0.005).

### The Proportion of Cardiovascular Change in Study Patients

SCFA concentrations, cardiovascular, and renal changes between index and following visits are presented in Table 4. The median follow-up time was 11.2 months (interquartile range, 8.5, 12.2 months). Among 65 patients who underwent carotid sonography, increased IMT was observed in 24.62% and increased PWV in 63.08%. Comparing the following visit with the index data, PWV increased by 0.18 m s^−1^ (*P* = 0.016). In 105 subjects, LV mass increased in 59.05%, LVMI increased in 35.24%, EF decreased in 55.24%, and eGFR decreased in 63.81%. Their eGFR decreased by 5.14 ml min^−1^ 1.73 m^−2^ (*P* = 0.001).

**Table 4:** SCFAs, cardiovascular and renal follow-up.

**The index and the difference between the index and the following data**		***P*-value**
Follow-up time (month)	11.2 (8.5, 12.2)	
***N*** **=** **65**		
**Carotid sonography**		
Index IMT (mm)	0.35 (0.30, 0.40)	
ΔIMT (mm)	0.01 ± 0.07	0.436
Increasing IMT (*n*)	16 (24.62%)	
Index PWV (m s^−1^)	4.30 (3.85, 4.80)	
ΔPWV (m s^−1^)	0.18 ± 0.60	0.016^*^
Increasing PWV (*n*)	41 (63.08%)	
***N*** **=** **105**		
Echocardiography		
Index LV mass (g)	65.50 (8.75, 107.25)	
ΔLV mass (g)	4.02 ± 18.07	0.015^*^
Increasing LV mass (*n*)	62 (59.05%)	
Index LVMI (g m^−2.7^)	32.88 (27.81, 39.96)	
ΔLVMI (g m^−2.7^)	−0.76 ± 7.38	0.101
Increasing LVMI (*n*)	37 (35.24%)	
Index EF (%)	67 (61, 71)	
ΔEF (%)	−1.19 ± 11.65	0.376
Decreasing EF (*n*)	58 (55.24%)	
Index eGFR (ml min^−1^ 1.73 m^−2^)	107.1 (88.25, 124.35)	
ΔeGFR (ml min^−1^ 1.73 m^−2^)	−5.14 ± 24.27	0.001^*^
Decreasing eGFR	67 (63.81%)	
**Short-chain fatty acid**		
Index AA (μM)	33.65 (18.58, 48.65)	
ΔAA (μM)	15.99 ± 61.04	0.015^*^
Index PA (μM)	2.00 (1.33, 3.28)	
ΔPA (μM)	−0.34 ± 2.94	0.276
Index BA (μM)	2.15 (1.23, 5.08)	
ΔBA (μM)	−2.27 ± 3.13	<0.001^*^

Data are expressed as median [25th, 75th], mean ± SD, or N (%).

* P < 0.05 between data from index and the following visits by pair t test.

*AA, acetate; BA, butyrate; EF, ejection fraction; eGFR, estimated glomerular filtration rate; IMT, intima-median thickness; LV, left ventricle; LVMI, left ventricular mass index; PA, propionate; PWV, pulse wave velocity*.

With regard to SCFAs, plasma acetate concentrations increased by 15.99 μM from 33.65 μM (*P* = 0.015). Plasma butyrate concentrations decreased by 2.27 μM from 2.15 μM (*P* < 0.001). Plasma propionate did not have significant changes between the index and the following years.

### Association of Worsened Blood Pressure With Changes in Kidney Function, Echographic Parameters and Plasma Short-Chain Fatty Acids

Comparison of the difference between the index and the following visit data is presented Table 5. No statistical eGFR change between the index and follow-up year was observed in the worsened BP group during this period (ΔeGFR in the worsened BP group, −11.47 ± 28.26 ml min^−1^ 1.73 m^−2^, *P* = 0.114). Compared with the index data, eGFR declined by 6.15 ml min^−1^ 1.73 m^−2^ in the stable BP group (*P* = 0.017). However, we observed that eGFR dropped by 11.42% in the worsened BP group, whereas no statistical change in eGFR was observed in stable BP in percentage (ΔeGFR in the worsened BP group, −11.42 ± 22.05%, *P* = 0.049; ΔeGFR in the stable BP group, −4.55 ± 16.32%, *P* = 0.062).

**Table 5:** The comparison between index and following visits in two groups.

	**Stable BP group** **difference**			**Worsened BP group** **difference**		
***N* = 65**	***N*** **=** **47**			***N*** **=** **18**		
	**Mean**	**SD**	***P*****-value**	**Mean**	**SD**	***P-*****value**
ΔeGFR (ml min^−1^ 1.73 m^−2^)	−6.15	17.10	0.017^*^	−11.47	28.26	0.114
ΔeGFR (%)	−4.55	16.32	0.062	−11.42	22.05	0.049^*^
ΔUPCR (mg g^−1^)	−47.23	1,468.85	0.826	1,306.67	6,005.49	0.369
ΔLDL-C (mg dl^−1^)	8.49	21.04	0.080	22.71	53.14	0.097
ΔTG (mg dl^−1^)	4.30	53.27	0.583	5.59	72.63	0.755
ΔUric acid (mg dl^−1^)	0.20	0.97	0.168	0.25	1.65	0.546
**Office BP**						
ΔSBP (mmHg)	0	11	0.873	7	14	0.048^*^
ΔDBP (mmHg)	0	12	0.799	7	14	0.039^*^
**ABPM profile**						
ΔMean SBP overall (mmHg)	−6	38	0.306	7	10	0.007^*^
ΔMean SBP awake (mmHg)	−8	35	0.111	9	9	0.001^*^
ΔMean SBP asleep (mmHg)	−7	31	0.118	4	15	0.286
ΔMean DBP overall (mmHg)	−4	19	0.174	5	6	0.003^*^
ΔMean DBP awake (mmHg)	−4	20	0.163	6	6	<0.001^*^
ΔMean DBP asleep (mmHg)	−3	17	0.170	2	8	0.237
ΔSBP load overall (%)	3.85	19.18	0.175	30.06	26.38	<0.001^*^
ΔSBP load awake (%)	2.87	16.21	0.231	33.00	25.14	<0.001^*^
ΔSBP load asleep (%)	4.49	27.00	0.260	21.50	34.59	0.017^*^
ΔDBP load overall (%)	2.70	9.19	0.050^*^	16.67	22.67	0.006^*^
ΔDBP load awake (%)	0.77	7.26	0.473	10.39	18.43	0.029^*^
ΔDBP load asleep (%)	6.72	18.03	0.014^*^	26.94	38.15	0.008^*^
**Carotid artery**						
ΔIMT (mm)	0.00	0.07	0.836	−0.02	0.08	0.310
ΔPWV (m s^−1^)	0.20	0.64	0.037^*^	0.14	0.51	0.268
**Cardiac echography**						
ΔLV mass (g)	2.04	17.93	0.444	5.92	27.53	0.374
ΔLVMI (g m^−2.7^)	−1.13	5.73	0.188	−0.13	7.61	0.945
ΔEF (%)	−3.21	12.71	0.090	4.67	11.96	0.116
**Plasma SCFA**						
ΔAA (μM)	22.75	28.63	<0.001^*^	6.12	115.31	0.830
ΔPA (μM)	−0.46	1.97	0.181	0.01	3.29	0.988
ΔBA (μM)	−2.12	2.54	<0.001^*^	−4.41	4.08	<0.001^*^

* P < 0.05 between data from index and the following visits by pair t test.

*AA, acetate; ABPM, ambulatory blood pressure monitoring; BA, butyrate; DBP, diastolic blood pressure; EF, ejection fraction; eGFR, estimated glomerular filtration rate; IMT, intima-median thickness; LDL-C, low-density lipoprotein cholesterol; LV, left ventricle; LVH, left ventricular mass; LVMI, left ventricular mass index; PA, propionate; PWV, pulse wave velocity; SBP, systolic blood pressure; TG, triglyceride; and UPCR, urine protein-to-creatinine ratio*.

In the worsened BP group, increase in almost every ABPM parameter was observed, such as mean SBP overall and awake, mean DBP overall and awake, and all BP load parameters (ΔSBP overall 7 mmHg, ΔSBP awake 9 mmHg, ΔDBP overall 5 mmHg, ΔDBP awake 6 mmHg, ΔSBP load overall 30.06%, ΔSBP load awake 33%, ΔSBP load asleep 21.50%, ΔDBP load overall 16.67%, ΔDBP load awake 10.39%, and ΔDBP load asleep 26.94%; all *P* < 0.05). Although ABPM profile revealed no BP change in the stable BP group, BP loads increased by 2.70 and 6.72% in DBP overall, and DBP asleep, respectively (both *P* < 0.05).

In echographic parameters, PWV increased by 0.2 m s^−1^ in the stable BP group, whereas there was no change in the worsened BP group (ΔPWV in the worsened BP group, 0.14 ± 0.51 m s^−1^, *P* = 0.268; ΔPWV in the stable BP group, 0.20 ± 0.64%, *P* = 0.037). Plasma acetate increased by 22.75 μM in stable BP groups, whereas no significant change was observed in worsened BP groups (ΔAA in the worsened BP group, 6.12 ± 115.31 μM, *P* = 0.831; ΔAA in the stable BP group, 22.75 ± 28.63 μM, *P* < 0.001). Plasma butyrate decreased by 2.12 μM in stable BP group (*P* < 0.001), whereas plasma butyrate decreased by 4.41 μM in worsened BP group (*P* < 0.001).

### Association Between Index Plasma Short-Chain Fatty Acids and Subclinical Cardiovascular Outcomes

The correlation between index plasma SCFAs and future cardiovascular changes and renal function outcome is presented in Table 6. One-micromolar increase in index plasma butyrate results in 1.381 times the ratio of having vs. not having worsened BP (*P* = 0.013). Additionally, the probability of getting vs. not getting decreasing EF was 1.281 times for each 1 μM increase in index plasma propionate (*P* = 0.046). These index plasma SCFAs remained significant after multivariable adjustment controlling for index data such as age, gender, BMI z-score, eGFR, and UPCR.

**Table 6:** The association between SCFAs and cardiovascular risks in children with early chronic kidney disease.

	**Index data**	**aOR**	***P*-value**
***N*** **=** **65**			
Worsened BP	AA (μM)	1.010	0.274
	PA (μM)	0.994	0.994
	BA (μM)	1.381	0.013^*^
Increasing IMT	AA (μM)	1.011	0.289
	PA (μM)	0.985	0.779
	BA (μM)	1.048	0.635
Increasing PWV	AA (μM)	1.007	0.491
	PA (μM)	1.431	0.080
	BA (μM)	0.885	0.149
***N*** **=** **105**			
Increasing LV Mass	AA (μM)	0.993	0.267
	PA (μM)	1.047	0.624
	BA (μM)	0.897	0.131
Increasing LVMI	AA (μM)	0.994	0.446
	PA (μM)	1.034	0.700
	BA (μM)	0.874	0.098
Decreasing EF	AA (μM)	0.993	0.252
	PA (μM)	1.281	0.046^*^
	BA (μM)	0.932	0.296
Decreasing eGFR	AA (μM)	1.006	0.417
	PA (μM)	1.012	0.809
	BA (μM)	0.935	0.489

* P < 0.05 in binary logistic regression adjusted for age, gender, BMI z-score, eGFR, and UPCR.

*aOR, adjusted odds ratio; BA, butyrate; BP, blood pressure; EF, ejection fraction; eGFR, estimated glomerular filtration rate; IMT, intima-median thickness; LV, left ventricle; LVMI, left ventricular mass index; AA, acetate; PA, propionate; and PWV, pulse wave velocity*.

## Discussion

An increase in plasma acetate levels was observed in stable BP group on follow-up, whereas there was no change in worsened BP group. This finding supported our hypothesis that the worsened BP group should have lower levels of plasma SCFA than the stable BP group, that is, acetate. Recent manipulations, which were expected to increase acetate intake in animal models, has shown lower BP. The infusion of acetate to cecum for 2 weeks can prevent obstructive sleep apnea-induced hypertension in rat model (15). High-fiber diet, considered to increase gut acetate production by lowering the *Firmicutes/Bacteroidetes* ratio and increasing the prevalence of *Bacteroides acidifaciens*, has been shown to reduce BP in mineralocorticoid excess-treated mice (16). In addition, acetate, a commonly used compound as dialysate, has been reported to lead to hypotension in hemodialysis patients and has been discontinued due to safety concerns (17). These results are consistent with our finding that acetate lowers BP. Acetate was reported to reduce BP, LVH, cardiac fibrosis, and renal fibrosis through downregulation of Egr1 in hypertensive mice (16). However, the receptors of acetate not totally protective for cardiovascular function are also reported. Olfr-78 (OR51E2 in humans), a mir-129-targeted receptor of SCFAs (mainly acetate and butyrate) expressed on the afferent arteriole, was reported to increase BP through mediating renin secretion and subsequent vasoconstriction (18).

We previously showed that higher concentrations of circulating propionate were found in children with CKD and non-CAKUT, who had a higher percentage of BP load at >25% or a nocturnal BP drop of <10% and worse renal function compared with those with CAKUT (8). However, in the present study, plasma propionate levels were not higher in the group with worse BP and there was no statistical change in plasma propionate levels at follow-up. However, plasma propionate was positively correlated with reduced F in the present study. Propionate has been demonstrated to possess anti-inflammatory effects such as the alleviation of cardiac hypertrophy, fibrosis, vascular dysfunction, and hypertension through T cell-dependent regulation in an angiotensin II-infused mouse model (19). However, Wang et al. reported that propionate interfered with fatty acid oxidation as a fuel resource for heart due to the lack of specific enzyme-utilizing short-chain (C3, C4) fatty acids in the propionate-perfused heart (20), suggesting that higher plasma propionate might cause adverse cardiac effects such as heart failure (20). This finding might explain the frequent association of cardiovascular dysfunction with dramatic increases in circulating concentrations of propionate or its metabolites in certain diseases, such as obesity, diabetes, and non-CAKUT renal diseases (compared with CAKUT) (8–10, 21).

The index plasma butyrate positively correlated with worse BP; however, we found that plasma butyrate levels were reduced in both the stable and worsened groups. The abundance of butyrate-producing bacteria and butyrate production by the gut microbiota were shown to exhibit significant and negative associations with BP in early pregnant women and adults with obstructive sleep apnea-induced hypertension (22, 23). Butyrate is reported to lower BP levels by inhibiting RAS (24) or by acting on Gpr41 (25). In this study, the initially higher baseline concentrations of plasma butyrate declined on follow-up; therefore, high baseline butyrate concentration may not be a good sign for future cardiovascular status but might instead indicate compensation for high BP. The decrease in plasma butyrate levels may be related to the deterioration of kidney function, that is, decline in eGFR. Wong et al. reported that serum concentrations of acetate, butyrate, and propionate were higher in healthy controls compared with patients with CKD including end-stage renal disease (26). Our study shows among the three SCFAs, plasma butyrate was the only one that declined in concentration on follow-up. Acetate, propionate, and butyrate were detected in the stool and colon, with an approximate molar ratio of 3:1:1 (27); however, this ratio in the colon and stool might change with a decline in eGFR levels, which requires the investigation of changes in SCFAs in stool and the gut microbiota between the baseline and follow-up.

This study is the first to elucidate the relationship of blood SCFAs with BP and other cardiovascular parameters in children and adolescents with CKD. Nevertheless, we acknowledge several limitations of our study. First, the study period was only about 1 year. Second, the analyses included the data collected only at two time points. Third, we did not include data on diet, probiotics, and antibiotics in our analyses. Finally, the gut microbiota was not analyzed in the present study.

The present study demonstrated that there was a difference in plasma acetate between subjects with and without hypertension and that plasma butyrate decreased on follow-up. However, the underlying mechanisms remain unclear. SCFAs are closely related to gut microbiota; therefore, agents which can potentially affect gut microbiota, including high-fiber diet, probiotics, and antibiotics, should be investigated in future studies. Cardiovascular changes, which are usually very mild in children with CKD, leads to serious issues in adulthood. Therefore, the findings of the current study should be confirmed in future investigations with long-term follow-up.

## Conclusion

We concluded that plasma acetate has a role in preventing from hypertension in children with CKD. Plasma propionate and butyrate seem to indicate the development of cardiovascular problems in our 1-year observation. Plasma butyrate shows a decrease in concentration, which is possibly attributable to the decline in eGFR. However, the role of circulating SCFAs in children with CKD still needs to be explored.

## Data Availability Statement

The original contributions presented in the study are included in the article/supplementary materials, further inquiries can be directed to the corresponding author/s.

## Ethics Statement

The studies involving human participants were reviewed and approved by the Institutional Review Board of Chang Gung Medical Foundation, Taipei, Taiwan (permit number 201601181A3). Written informed consent to participate in this study was provided by the participants' legal guardian/next of kin.

## Author Contributions

C-NH, P-CL, and Y-LT conceived and designed the experiments. Y-LT contributed reagents and materials. I-CL, M-HL, and P-CL performed carotid echography and echocardiography. C-NH and P-CL analyzed the data. P-CL wrote the manuscript. M-YY was the thesis advisor. All authors contributed to the article and approved the submitted version.

## Conflict of Interest

The authors declare that the research was conducted in the absence of any commercial or financial relationships that could be construed as a potential conflict of interest.
